# Bilateral dislocation of the hip joint and associated pathological changes in the ossa coxae and femora of a European roe deer (*Capreolus capreolus*)

**DOI:** 10.1371/journal.pone.0290586

**Published:** 2023-08-24

**Authors:** Uwe Kierdorf, Stefan Flohr, Christian Dullin, Horst Kierdorf

**Affiliations:** 1 Department of Biology, University of Hildesheim, Hildesheim, Germany; 2 Institute for Diagnostic and Interventional Radiology, University Medical Center Göttingen, Göttingen, Germany; University of Szeged Institute of Biology: Szegedi Tudomanyegyetem Biologia Intezet, HUNGARY

## Abstract

We describe a bilateral craniodorsal dislocation of the hip joint in a free-ranging young roe buck and the associated pathological changes in the ossa coxae and femora of the animal. The highly symmetrical dislocation, which is considered to have developed secondary to hip dysplasia, caused the formation of two false acetabula that each consist of several, partially fused bone portions. The femora exhibit symmetrical outgrowths that extend from the greater trochanter along the intertrochanteric crest to the lesser trochanter. Formation of these outgrowths is attributed to abnormal traction at muscle attachment sites due to the displacement of the femora. On radiographic examination, both femora show signs of avascular necrosis in their head regions and of fatty marrow necrosis in their shafts, which is attributed to the damage of the arterial blood supply of the femora that was associated with the dislocation. The fact that, according to the hunter who shot the buck, the animal’s locomotion was inconspicuous suggests that the false hip joints functioned quite well, thereby demonstrating a remarkable capacity of the musculoskeletal system for functional recovery.

## Introduction

The coxofemoral (hip) joint is formed by the acetabulum of the hip bone (os coxae) and the head of the femur (caput ossis femoris). In mammals, the acetabulum is deepened by a well-developed ring of fibrocartilage, the acetabular lip (labrum acetabulare), which is attached to its bony rim (margo acetabuli) [1]. The hip joint is surrounded by a spacious joint capsule and thickly covered by muscles.

The term dislocation describes a condition in which the contact between bones that normally articulate has been completely lost [2, 3]. The term luxation is also often used for this condition [4–6], while a partial loss of contact between articulating bones is referred to as subluxation [2, 3, 6]. Dislocation of the hip joint can be caused by major trauma, but can also occur secondary to hip dysplasia. Hip dislocation is frequently associated with a partial or complete rupture of the ligament of the head of the femur (ligamentum capitis ossis femoris) or an avulsion fracture at the ligament’s attachment site, damage to the acetabular lip, tearing of the joint capsule and injury to surrounding soft tissues [6, 7].

Hip dysplasia is a congenital, bilaterally occurring condition characterized by a lack of conformity between the femoral head and the acetabulum [5, 8]. A common finding in hip dysplasia is an abnormally shallow acetabulum, which causes joint laxity and leads to degenerative joint disease (DJD). Among domestic mammals, hip dysplasia is most commonly observed in dogs, especially large breeds, but the condition also occurs in other domesticated animals [5].

Hip dysplasia is a well-known predisposing factor for hip dislocation, which can develop spontaneously due to the inherent instability of the joint or following a mechanical impact that in a normally developed joint would not lead to a displacement of the femoral head from the acetabulum [2]. In domestic mammals, hip dislocations are mostly craniodorsal, i.e., the dislocated femoral head is positioned craniodorsal to the acetabulum [4, 6, 9]. In the case of a long-standing dislocation, the pressure by the femoral head against the hip bone can cause the formation of a secondary (false) acetabulum or neo-acetabulum [2, 10–12].

Compared to domesticated animals, hip dislocation has only rarely been reported from deer [13, 14] and other wild mammals, e.g. chimpanzees (*Pan troglodytes*) [15], and information on the frequency of the condition in mammalian wildlife is largely missing. The present paper describes a unique case of bilateral dislocation of the hip joint in a young male European roe deer (*Capreolus capreolus*) that had caused the formation of highly symmetrical pathological changes in both ossa coxae and the proximal portions of both femora.

## Materials and methods

We studied the hip bones and femora of a young roe buck that had been taken in 1999 in the course of regular hunting operations in the state-owned forest district Königsforst near Cologne (federal state of North Rhine-Westphalia, Germany). The European roe deer is listed as a huntable species under both the German federal hunting law and the state hunting law and not subject to further species protection. Therefore, no additional permits were required for obtaining the specimen under study. The largely macerated and dried hip bones and femora of the roe buck were originally submitted to the Research Station of Wildlife Biology of the federal state of North Rhine-Westphalia (FJW-NRW; Bonn, Germany) by the state forest officer in charge of the area, and from there forwarded to the first author for examination. According to observations prior to culling, locomotion of the roe buck was inconspicuous. During evisceration, the buck’s ossa coxae were split in the symphyseal region and the dorsal portion of the wing of the ilium (ala ossis ilii) was cut off bilaterally. No further skeletal material from the animal was available.

For comparison with the pathological bone specimens, we also studied the ossa coxae (fused in the symphyseal region) and femora of an adult female roe deer (collection number of the skeleton: 3945) from the osteological reference collection at the Center for Baltic and Scandinavian Archeology (Schleswig, Germany). Henceforth, these bones are referred to as “controls”.

The bones analyzed in this study originated from animals killed during legal hunting operations for population management. As the individuals had not been killed for the purpose of this study, ethical approval was not required.

The hip bones and femora of the male and the female roe deer were photographed with a digital camera (Canon EOS 80D) and radiographed using a digital X-ray apparatus (Philips DigitalDiagnost, 70 kV). In addition, the pathological femora of the buck were scanned using an in-vivo microCT-scanner (QuantumFX, Perkin Elmer) operated at 90 kV and 200 μA (FOV 73 × 73 mm). The acquired images were processed using Adobe Photoshop. Following image acquisition, the left femur of the buck was transversely sectioned in its distal portion and the object located in the medullary cavity was removed. A transverse slice from this object was subsequently analyzed by multipoint laser-induced breakdown spectroscopy (LIBS) using a Keyence EA-300 elemental analyzer attached to a Keyence VHX-7000 microscope.

## Results

### Hip bones

**Fig 1:**
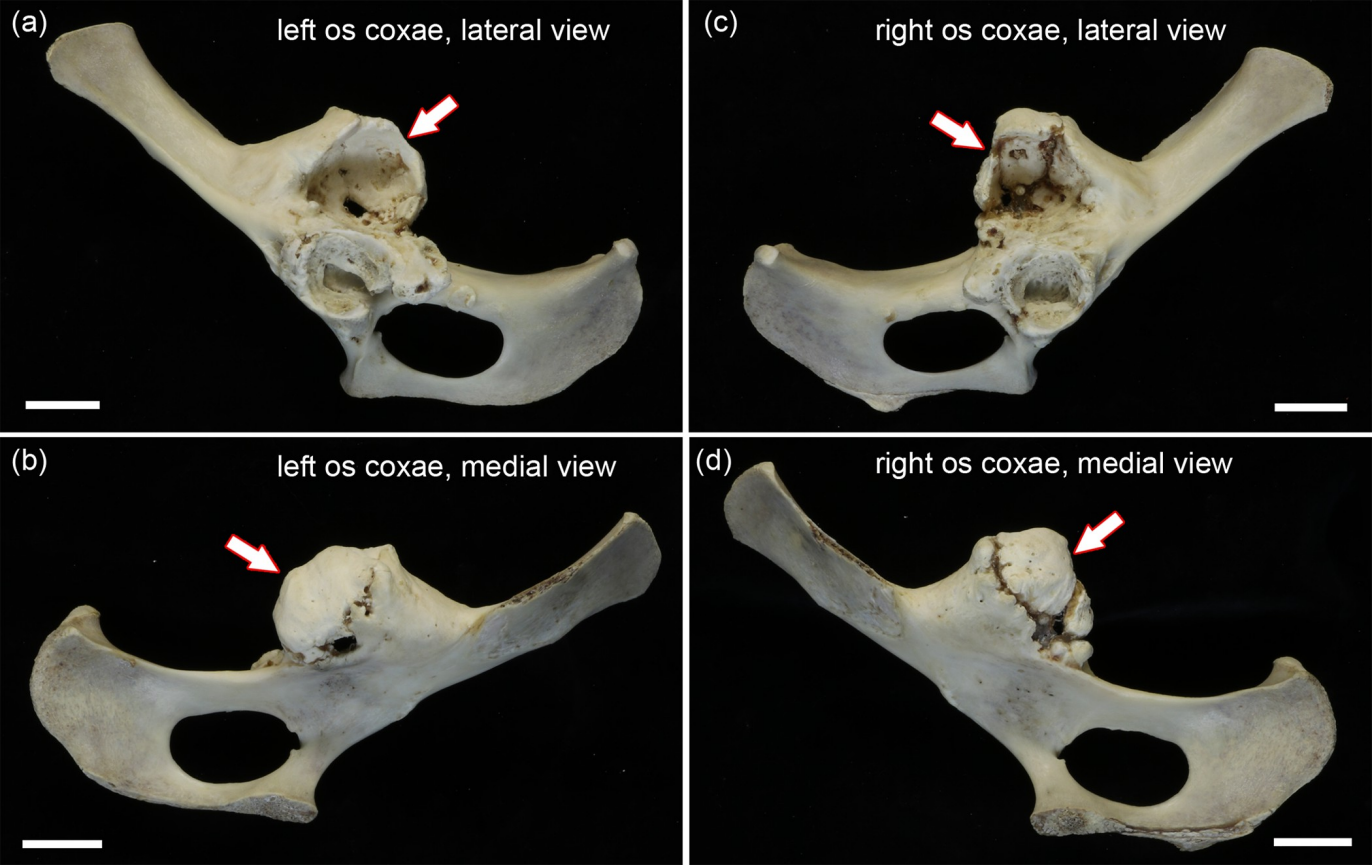
Overview images of pathological hip bones of the roe buck; bars equal 20 mm. **(a)** Left os coxae, lateral view. **(b)** Left os coxae, medial view. **(c)** Right os coxae, lateral view. **(d)** Right os coxae, medial view. Arrows: false acetabula.

**Fig 2:**
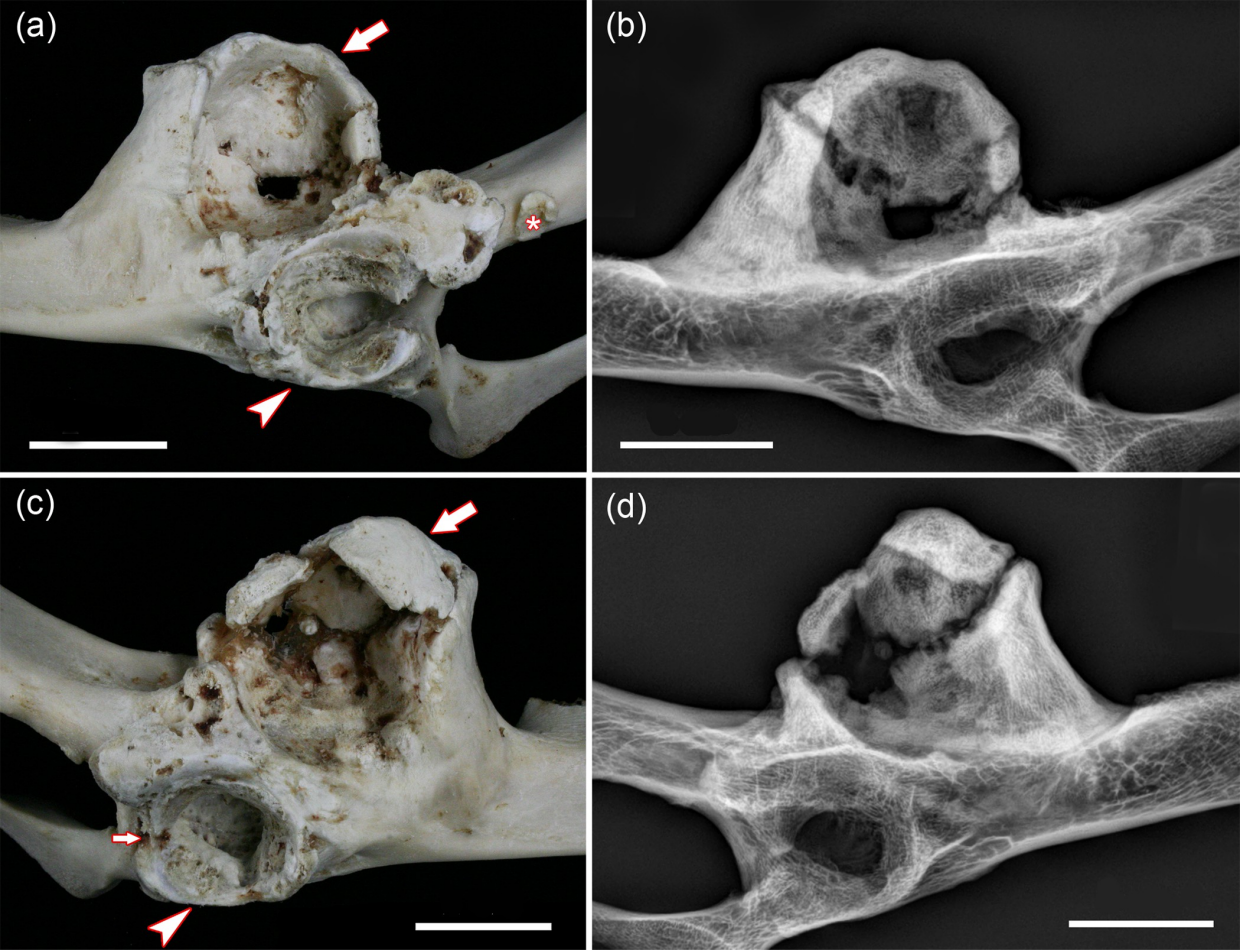
True and false acetabula of the ossa coxae in the roe buck; bars equal 20 mm. **(a)** Left os coxae, lateral view; arrowhead: true acetabulum, arrow: false acetabulum, asterisk: isolated osteophyte on the ischium; anterior to the left of the image**. (b)** Radiograph (lateromedial projection) of left os coxae; note composite structure of false acetabulum. **(c)** Right os coxae, lateral view; arrowhead: true acetabulum, large arrow: false acetabulum, small arrow: bony bridging of acetabular notch; anterior to the right of the image. **(d)** Radiograph (lateromedial projection) of right os coxae; note composite structure of false acetabulum.

The ossa coxae of the roe buck show a number of highly symmetrical pathological changes (Figs 1A–1D and 2A–2D). Both acetabula appear more narrow and shallow than those of the (control) female roe deer (S1 Fig). Due the extensive pathological changes in both acetabula of the roe buck, it was, however, not possible to unambiguously reconstruct their original dimensions. Both acetabula exhibit an irregularly thickened rim with formation of broad-based osteophytes, and an uneven structure of the facies lunata with erosions and pitting as well as deposition of new bone (Fig 2A–2C). In the right hip bone, the acetabular notch is bridged by a bony outgrowth. Moreover, some deposition of new bone has occurred in the craniodorsal part of the right acetabular fossa (Fig 2C). The left os coxae exhibits a small osteophyte caudal to the acetabulum (Fig 2A).

The most conspicuous pathological change in both ossa coxae is the presence of a newly formed joint socket (false acetabulum) that is located craniodorsal to the acetabulum in the region of the spina ischiadica (Figs 1A–1D and 2A–2C). The false acetabula are larger and deeper than the true acetabula, and each consists of several portions that have partially fused. The presence of non-ossified gaps between the different components (Fig 2B–2D) reveals that this fusion was incomplete at the time of death. The gaps were bridged by soft (unmineralized) tissue, remnants of which are still present in the macerated specimens.

### Femora

**Fig 3:**
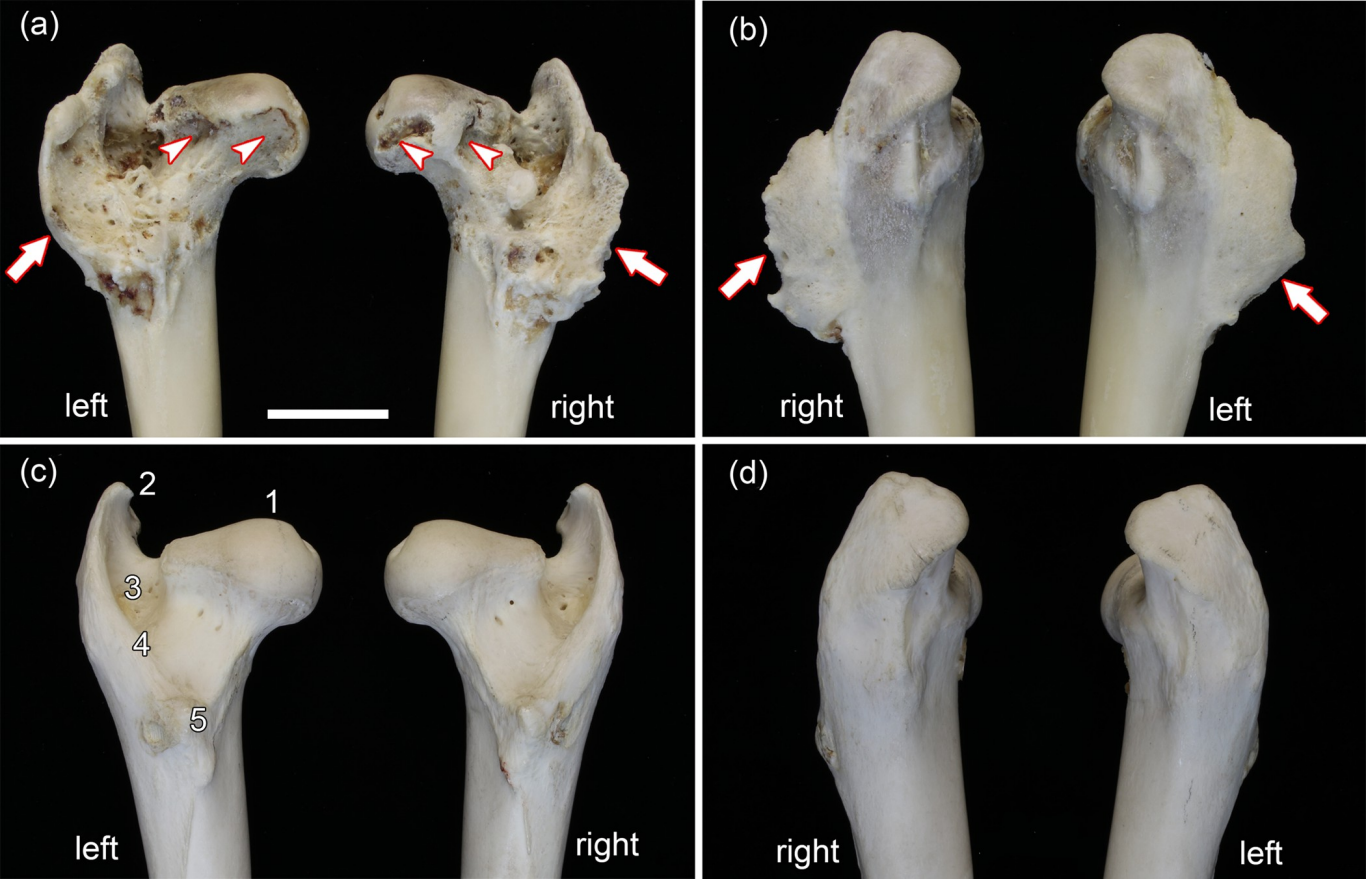
Proximal regions of the pathological femora of the roe buck (a, b) and the control femora of the female roe deer (c, d); bar in (a) equals 20 mm and applies to all images. **(a)** Caudal views of pathological femora. **(b)** Cranial views of pathological femora. **(c)** Caudal views of normal (control) femora. **(d)** Cranial views of normal (control) femora. Note flattening, eburnation and defects (arrowheads) of the heads, exostoses in the trochanteric fossa, and outgrowth (arrows) in the region of the crista intertrochanterica of the pathological femora. 1: Caput ossis femoris. 2: Trochanter major. 3: Fossa trochanterica. 4: Crista intertrochanterica. 5: Trochanter minor.

**Fig 4:**
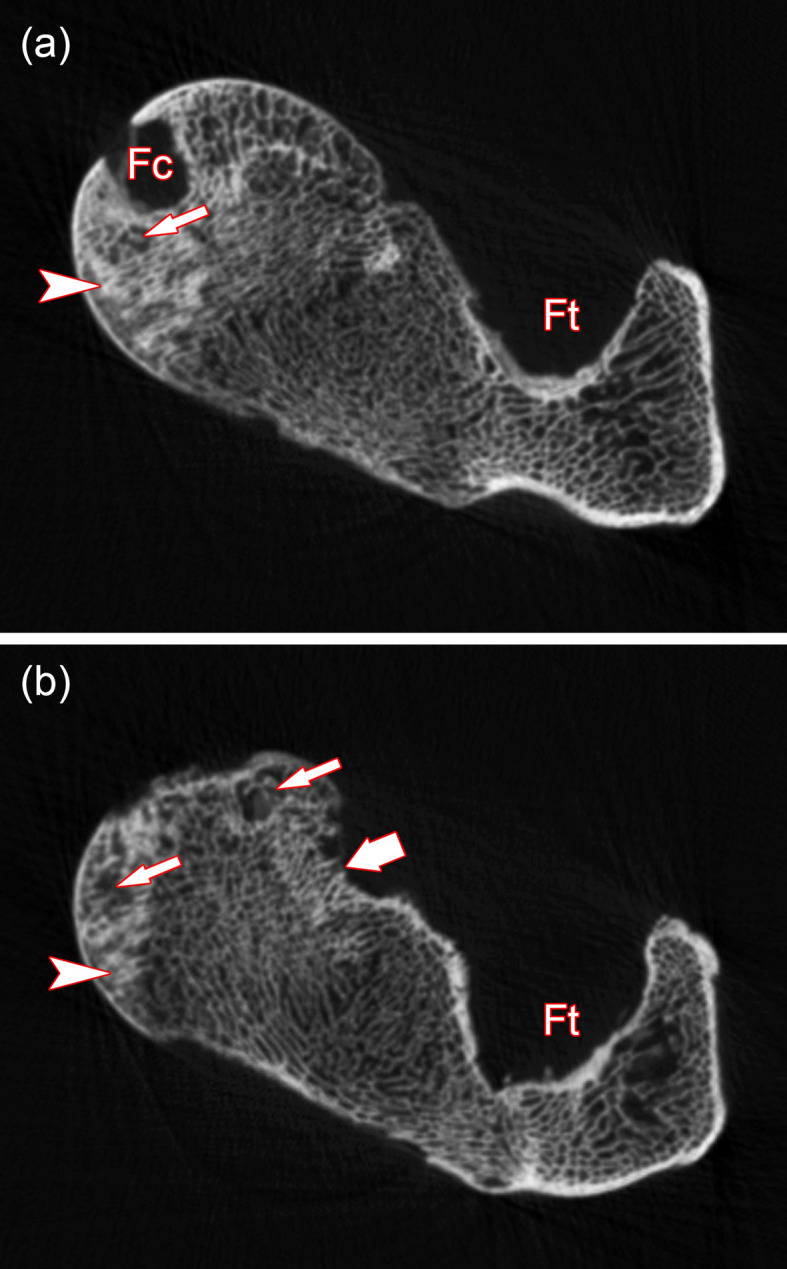
CT-images (transverse slices) of the proximal portion (femoral head region) of the right pathological femur of the roe buck. **(a)** Slice located in the region of the fovea capitis ossis femoris (Fc). **(b)** Slice located further distally. Ft: fossa trochanterica, arrowheads: areas of bone sclerosis, thin arrows: lucent areas indicative of bone resorption, thick arrow: bone defect.

The proximal portions of the pathological and the control femora are shown in Fig 3A–3D. Like in the hip bones, also in the femora of the roe buck, the pathological changes are of a highly symmetrical nature. The heads of the pathological femora are more flattened than in the controls (Fig 3A–3C), show eburnation medially (around the fovea capitis femoris) and exhibit larger bone defects medioventrally and caudoventrally (Fig 3A and 3B). CT imaging demonstrated the co-occurrence of sclerotic and lucent areas in the femoral heads (Fig 4A and 4B). In the right femur, smaller defects of the bone surface are present craniodorsally at the transition between femoral head and neck.

**Fig 5:**
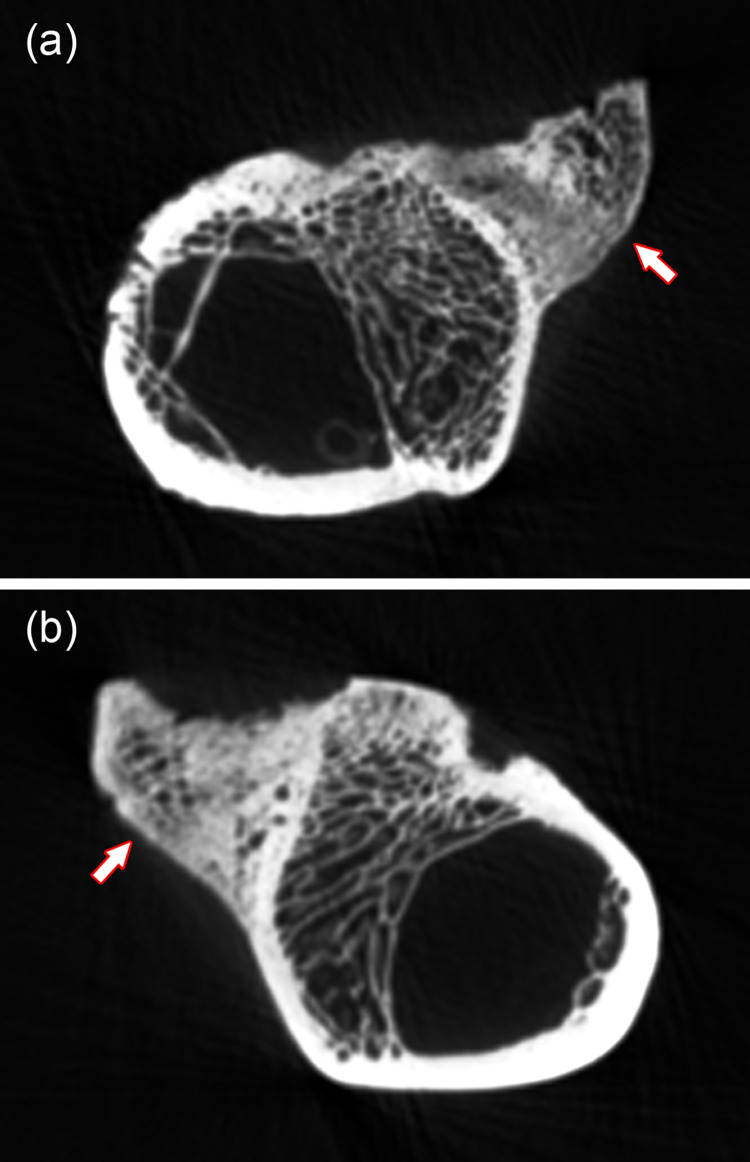
CT-images (transverse slices) of the pathological femora (proximal portion, below femoral head). **(a)** Right femur. **(b)** Left femur. Arrows: Bony outgrowths in the region of the intertrochanteric crest.

In both pathological femora, an elongated flattened (laminar) outgrowth extends from the caudal margin of the greater trochanter along the intertrochanteric crest to the lesser trochanter (Fig 3A and 3B). The surface structure of these outgrowths is more porous than that of the adjacent bone. Their lateral surface is convex, the medial one concave. The caudal border of both outgrowths is wavy, with some short, spike-like protuberance in the right femur. CT-imaging demonstrated the predominantly trabecular nature of the outgrowths (Fig 5A and 5B). Formation of the bony outgrowth in this region is attributed to abnormal traction at muscle attachment sites (musculus gluteus medius and musculus quadriceps femoris) due to the dislocation.

The surface of the intertrochanteric fossa of both pathological femora is uneven with bony projections (exostoses) that are more pronounced in the right femur (Fig 3A and 3B). Bilaterally, the femoral head does not fit well into the true acetabulum as the caput is too large for the fossa. In contrast, the spacious false acetabula easily accommodate the femoral heads.

**Fig 6:**
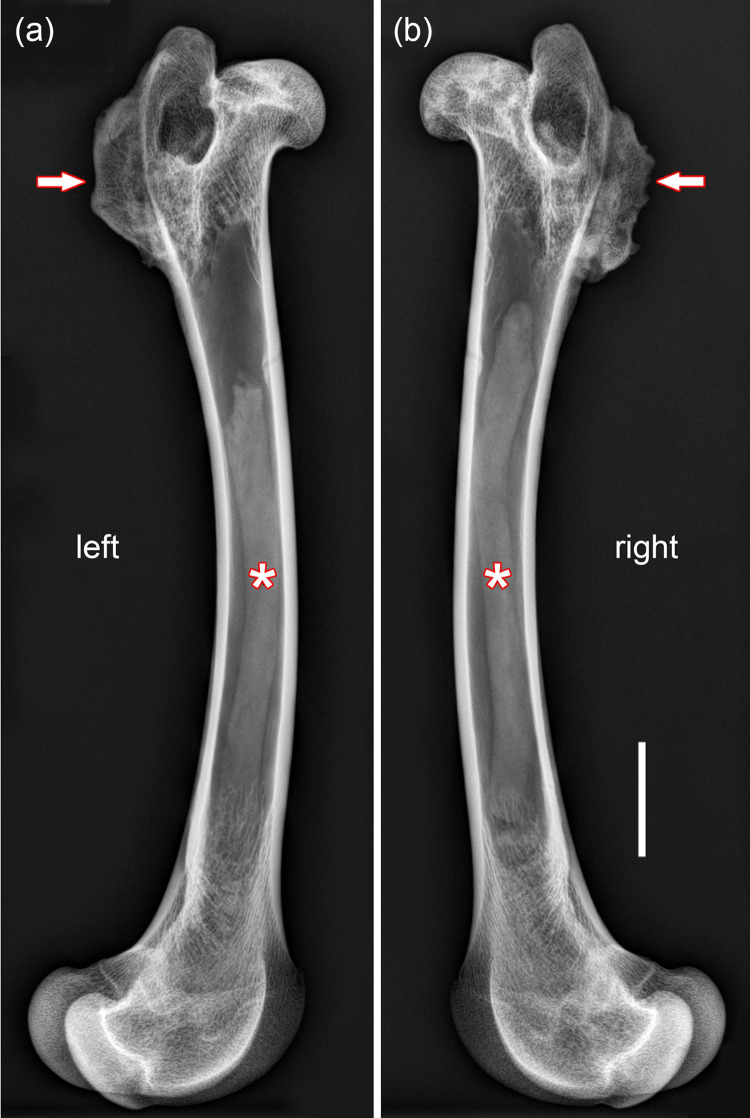
Radiographs (mediolateral projections) of the pathological femora of the roe buck; bar in (b) equals 20 mm and applies to both images. **(a)** Left femur. **(b)** Right femur. Arrows: Bony outgrowths in the regions of the intertrochanteric crest. Asterisks: Objects in marrow cavity.

**Fig 7:**
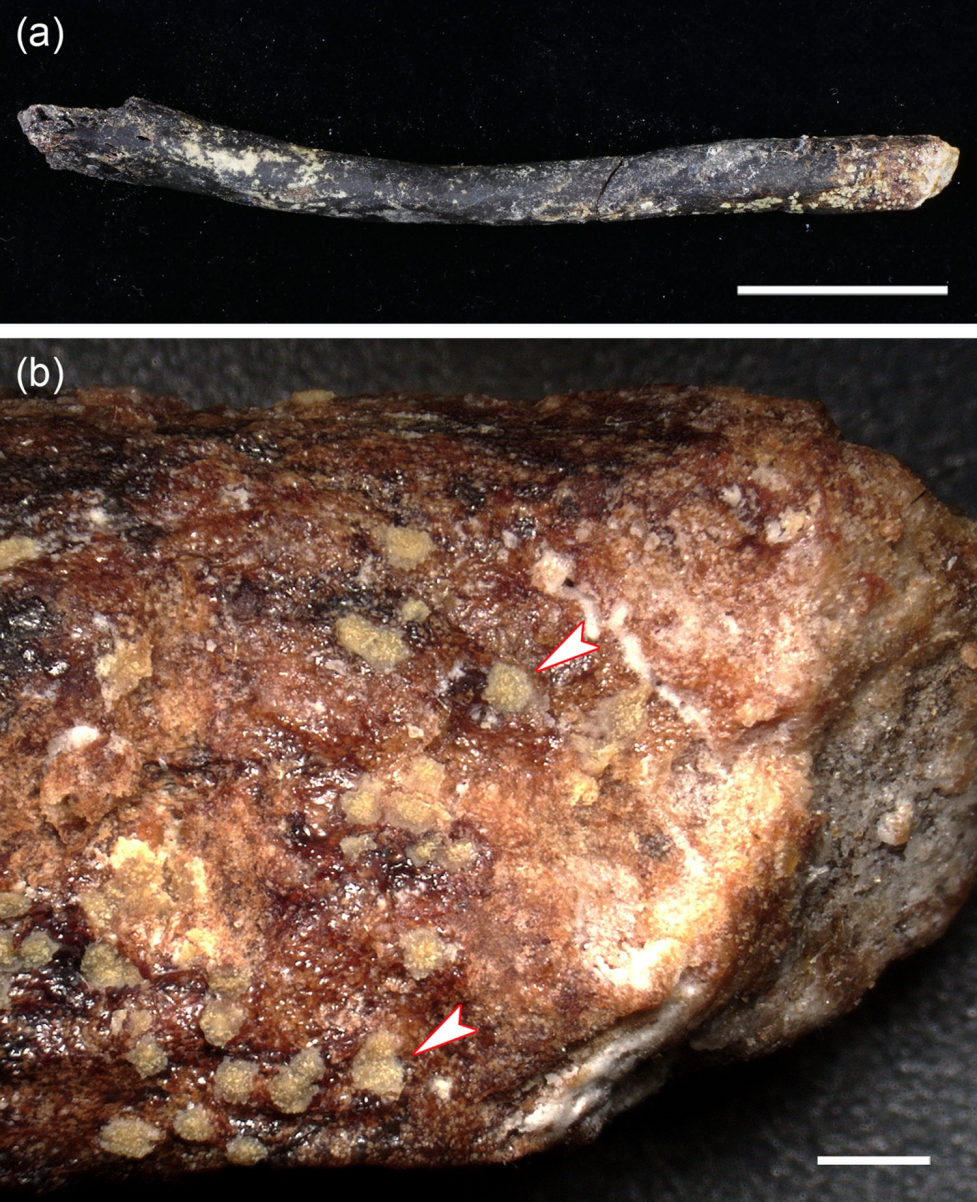
Object removed from the marrow cavity of the left femur of the roe buck. **(a)** Overview image of the object that is composed of organic material; the bar equals 20 mm. **(b)** Detail of object whose surface exhibits yellowish plaques (arrowheads) consisting of fat; bar equals 1 mm.

Radiographic examination showed the presence of an elongated (freely movable) object of low radiodensity in the medullary cavity of both pathologic femora (Fig 6A and 6B). In the left femur, the object was removed from the medullary cavity for analysis. It exhibits a dark-brown color and homogeneous structure (Fig 7A), and its surface is covered with numerous yellowish plaques identified as fat (Fig 7B). Multipoint analysis of a transverse slice of the object by LIBS showed the presence of carbon, oxygen, and hydrogen, but not of calcium or phosphorus. Mean (± SD) concentrations (weight %) from the analysis of six sampling spots from a cross-sectional slice were 70.1 ± 4.37 for carbon, 22.2 ± 4.57 for oxygen, and 7.7 ± 0.69 for hydrogen. This and the low radiodensity of the object indicates that it is entirely composed of non-mineralized organic material, which we consider to represent dried and condensed remnants and/or breakdown products of fatty marrow.

## Discussion and conclusions

The case described here represents an example of bilateral craniodorsal hip dislocation and associated pathological changes in both hip bones and femora of a free-ranging roe buck. To the best of our knowledge, no such case of highly symmetrical pathological changes in the ossa coxae and femora has previously been reported in roe deer or other cervid species. The bilateral nature of the lesions strongly favors the interpretation that the condition developed secondary to congenital hip dysplasia. The bilateral craniodorsal dislocation of the hip joint had led to the formation of a false acetabulum in both ossa coxae.

In humans, various attempts have been made to classify congenital hip disease (developmental dysplasia of the hip) according to its severity. In adults, Hartofilakidis et al. [16] distinguished between (i) dysplasia (Type A, femoral head contained within the original (true) acetabulum), (ii) low dislocation (Type B, femoral head articulates with a false acetabulum that partly covers the true acetabulum), and (iii) high dislocation (Type C, femoral head located completely out of the true acetabulum). The latter type was further separated into two subtypes, *viz*. C1 when the femoral head articulates with a false acetabulum, and C2 when there is no false acetabulum. Applying these criteria to the present (non-human) case, the condition in the roe deer would be classified as a bilateral C1 case. Applying the 5-grade classification scheme of canine hip dysplasia by the FCI (Fédération Cynologique Internationale), the condition would be classified as the most severe grade E [17].

The marked osteophytosis along the acetabular rim precluded detailed measurements of the width and depth of the true acetabula in the roe buck. However, visual comparison with the control specimens suggests their abnormal shallowness, and it is concluded that this was the predisposing factor for the bilateral joint dislocation.

The dislocation must have been a long-standing condition, since bilaterally a deep false acetabulum had formed that consisted of several, partially fused portions. Bilateral formation of a false acetabulum is considered the consequence of a constant irritation of the periosteum of the hip bone by the femoral head and the formation of larger quantities of new bone in response to this stimulus [11]. It has been reported that the newly formed joint socket can become lined by cartilage, and that even a structure resembling a joint capsule can secondarily develop around the “new joint” [11]. Formerly, the term nearthrosis was used for these “new joints” formed in the case of long-standing dislocations [10, 11].

Displacement of the femoral head from the true acetabulum must have been associated with severe damage to the acetabular lip and the ligaments of the hip, injury to the joint capsule with loss of synovial fluid, and lesions in surrounding soft tissues [6, 7].

Some of the pathological changes seen in the ossa coxae and femora, *viz*., osteophyte formation around the acetabular margin and eburnation of the femoral head, correspond to those typically seen in the case of DJD [5, 18, 19]. It is possible that these lesions developed or started to develop during an intermediate stage of instability and subluxation of the dysplastic hip joints that preceded the dislocation [8]. However, as only the final outcome of the pathological process could be studied, such a course of events cannot be proven. The lesions in the ossa coxae and femora of the roe buck could also have developed in the case of an “instant” dislocation, and associated damage to the joint, during early life without an intermediate stage of subluxation.

It is suggested that the bilateral hip joint dislocation in the roe buck was associated with severe damage to the arterial vessels supplying the femora, causing traumatic osteonecrosis in the femoral head and shaft due to a disrupted blood supply. Evidence for avascular necrosis in the femoral heads is the co-occurrence of sclerotic and lucent bone lesions, which are considered characteristic for osteonecrosis [20]. The lucent areas are indicative of localized bone resorption, while sclerosis is attributed to either reparative bone formation or dystrophic calcification of necrotic bone areas [20].

The two loose objects present in the marrow cavities of the pathological femora were diagnosed as dried and condensed remnants and/or breakdown products of fatty marrow, thus indicating a case of “mummified marrow necrosis” [21]. Because of its sparse blood supply (terminal vascularization, poor capillary network), fatty marrow is more at risk of necrosis than red marrow with its nonterminal vascularization and rich blood supply [20, 21]. In humans, necrosis of fatty marrow is considered a frequent lesion that can affect any bone segment of the appendicular skeleton, with mechanical interruption of arteries due to physical injury as one of the possible causes [21].

In conclusion, the pathological changes present in the ossa coxae and proximal femora of the roe buck are diagnosed as the result of a bilateral craniodorsal hip joint dislocation that most likely developed secondary to congenital hip dysplasia. The long-standing condition had caused the formation of two large false acetabula, each of which is composed of several, partially fused bone. Formation of a laminar bony outgrowth in the proximal region of both femora is attributed to abnormal muscle traction resulting from the hip joint dislocation. According to our interpretation, the dislocation was associated with severe damage to the arterial vessels supplying the femora, causing osteonecrosis in the femoral head and shaft.

A limitation of the study is the fact that only macerated dry bones were available for study. However, in our view a reliable diagnosis of the pathological condition in the roe buck is nevertheless considered possible, based on the detailed analysis of the lesions present in its hip bones and femora. The observation that locomotion of the buck was inconspicuous suggests that the false hip joints functioned quite well, thereby demonstrating a remarkable capacity of the musculoskeletal system for functional recovery. Survival of the buck may have been favored by the fact that large predators were not present in the area.

## Supporting information

S1 Fig Normal ossa coxae (control) of a female roe deer; bars equal 20 mm. (a) Left lateral view, (b) Dorsal view. (c) Radiograph (dorsoventral projection). 1: Tuber coxae. 2: Tuber sacrale. 3: Spina ischiadica. 4: Tuber ischiadicum. 5: Margo acetabuli. 6: Fossa acetabuli. 7: Symphysis pubica. 8: Foramen obturatum. 9: Arcus ischiadicus.
(TIF)
