# Supplementary figures and images for: Bilateral dislocation of the hip joint and associated pathological changes in the ossa coxae and femora of a European roe deer (Capreolus capreolus)
Source: PLoS One. 2023 Aug 24;18(8):e0290586. doi: 10.1371/journal.pone.0290586 (PMC10449113; doi:10.1371/journal.pone.0290586)

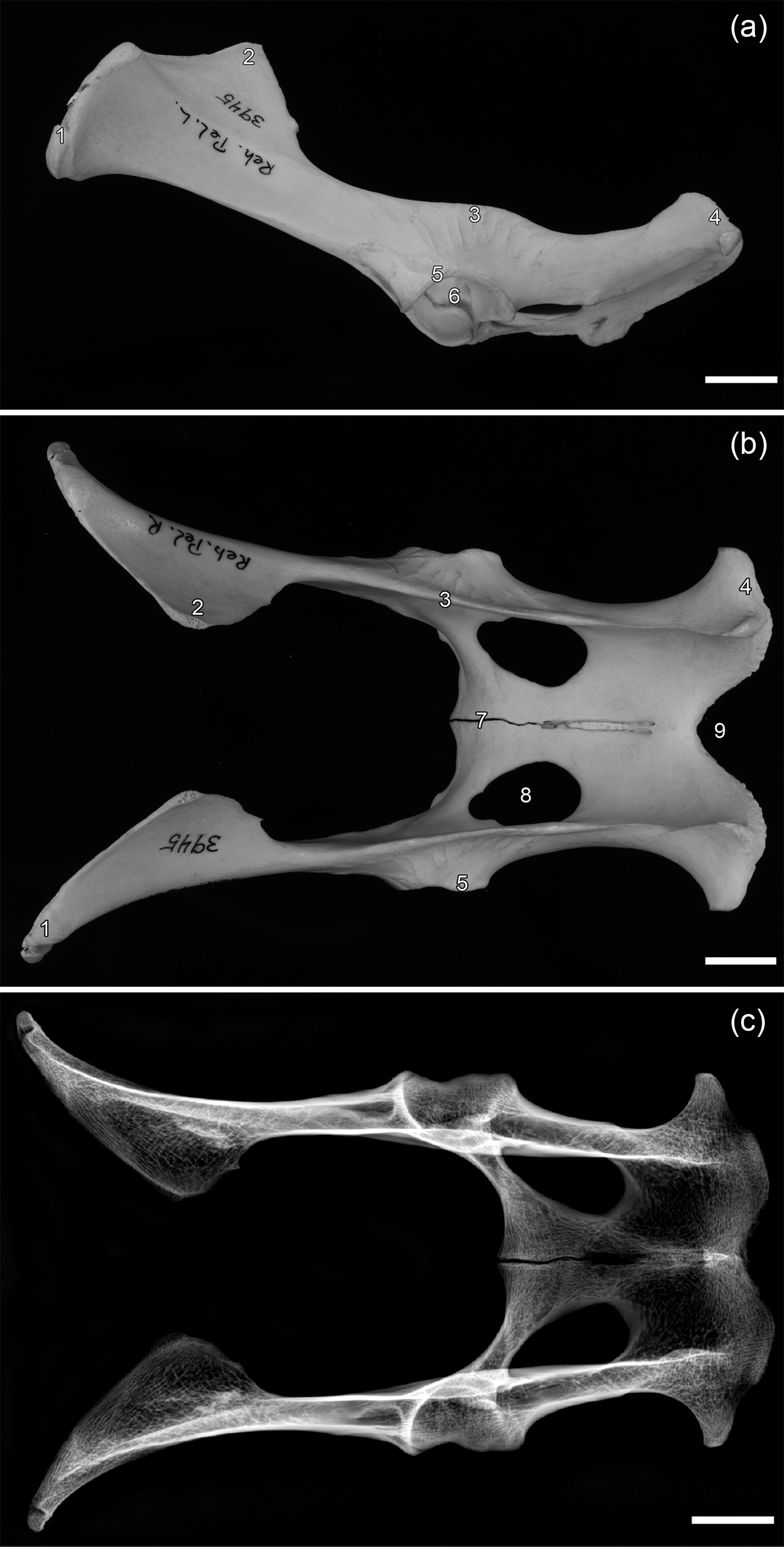

Supplement: S1 Fig — (a) Left lateral view, (b) Dorsal view. (c) Radiograph (dorsoventral projection). 1: Tuber coxae. 2: Tuber sacrale. 3: Spina ischiadica. 4: Tuber ischiadicum. 5: Margo acetabuli. 6: Fossa acetabuli. 7: Symphysis pubica. 8: Foramen obturatum. 9: Arcus ischiadicus. (TIF) [file pone.0290586.s001.tif]
